# Deficits in skilled motor and auditory learning in a rat model of Rett syndrome

**DOI:** 10.1186/s11689-020-09330-5

**Published:** 2020-09-28

**Authors:** Katherine S. Adcock, Abigail E. Blount, Robert A. Morrison, Amanda Alvarez-Dieppa, Michael P. Kilgard, Crystal T. Engineer, Seth A. Hays

**Affiliations:** 1 School of Behavioral and Brain Sciences, The University of Texas at Dallas, 800 West Campbell Road, Richardson, TX 75080-3021 USA; 2 Texas Biomedical Device Center, The University of Texas at Dallas, 800 West Campbell Road, Richardson, TX 75080-3021 USA; 3 Department of Bioengineering, Erik Jonsson School of Engineering and Computer Science, The University of Texas at Dallas, 800 West Campbell Road, Richardson, TX 75080-3021 USA

**Keywords:** Rett syndrome, MeCP2, Motor, Auditory, Learning

## Abstract

**Background:**

Rett syndrome is an X-linked neurodevelopmental disorder caused by a mutation in the gene *MECP2*. Individuals with Rett syndrome display developmental regression at an early age, and develop a range of motor, auditory, cognitive, and social impairments. Several studies have successfully modeled some aspects of dysfunction and Rett syndrome-like phenotypes in transgenic mouse and rat models bearing mutations in the *MECP2* gene. Here, we sought to extend these findings and characterize skilled learning, a more complex behavior known to be altered in Rett syndrome.

**Methods:**

We evaluated the acquisition and performance of auditory and motor function on two complex tasks in heterozygous female *Mecp2* rats. Animals were trained to perform a speech discrimination task or a skilled forelimb reaching task.

**Results:**

Our results reveal that *Mecp2* rats display slower acquisition and reduced performance on an auditory discrimination task than wild-type (WT) littermates. Similarly, *Mecp2* rats exhibit impaired learning rates and worse performance on a skilled forelimb motor task compared to WT.

**Conclusions:**

Together, these findings illustrate novel deficits in skilled learning consistent with clinical manifestation of Rett syndrome and provide a framework for development of therapeutic strategies to improve these complex behaviors.

## Background

Rett syndrome is a rare neurodevelopmental disorder associated with a mutation in the X-linked gene *MECP2*. This disorder mostly affects females, who display normal early development followed by regression of already acquired skills [4]. Individuals with Rett syndrome exhibit a range of symptoms, including seizures, breathing abnormalities, motor impairments, changes in sensory responses, anxiety, and speech-language deficits [4, 14]. The development of interventional strategies to improve function in these domains is of clear clinical importance.

To address this and provide a testbed for evaluation, transgenic mouse and rat models bearing mutations in the *MECP2* gene have been developed [12, 13, 25, 31]. Some features of these models have been well characterized and provide substantial insight into the pathophysiology of Rett syndrome. However, testing complex behaviors in *Mecp2* rodents is in its early stages. Initial work successfully modeled psychomotor regression similarly seen in girls with Rett syndrome by using a seed opening task in *Mecp2* rats [31]. Other studies using the same rat model have found abnormalities in gait, rotarod performance, and speech discrimination performance [3, 9]. It is likely that detailed characterization of other complex behaviors known to be altered in Rett syndrome, such as skilled learning, could expand the utility of these animal models. Here, we sought to characterize the acquisition and performance of skilled motor function and to replicate the previous findings characterizing auditory discrimination performance.

To do so, we trained female heterozygous *Mecp2* rats and wild-type (WT) littermate control rats to perform a speech discrimination task to assess auditory processing or a skilled forelimb reaching task to evaluate motor function. In both the complex auditory and motor tasks, *Mecp2* rats exhibit acquisition of the task and improved performance with continued training. However, the rate of learning, as well as several metrics of performance, are reduced in *Mecp2* rats compared to WT, indicative of a deficit in skilled learning. Impaired performance corresponds to dysregulated neural function in auditory and motor networks in transgenic *Mecp2* models [9, 21]. These results provide a novel characterization of skilled learning in *Mecp2* rats and develop a framework for future studies to evaluate the effect of potential interventions on complex behavioral tasks.

## Methods

### Subjects

Twenty-four female *Mecp2* heterozygous rats and 18 female wild-type littermates were used in this study. Rats were generated by breeding a *Mecp2* heterozygous Sprague-Dawley female with a wild-type Sprague-Dawley male. Female breeders were obtained from Horizon Discovery (SAGE labs), using zinc finger nuclease technology that generated a 71 base pair deletion in exon 4 [3, 9, 31]. Experimenters were blind to the genotype. Animals underwent behavioral assays and training between 4–8 months of age. Nine *Mecp2* rats and 11 wild-type rats underwent auditory discrimination training. Fifteen *Mecp2* rats and 13 wild-type rats underwent motor training. Following motor training, 7 of the *Mecp2* rats and 9 wild-type littermates underwent intracortical microstimulation (ICMS). All animals were housed in a 12:12 h reversed light-dark cycle and were food-deprived during training. A small subset of animals did not complete all tasks they were assigned to (Additional file 1). Similar to previous studies [9], 18 of the 24 *Mecp2* rats had one or more seizures. When seizures occurred, rats were given a 30-min break or removed from the session for the day. All protocols were approved by The University of Texas at Dallas Institutional Animal Care and Use Committee.

### Auditory behavioral testing

**Fig. 1:**
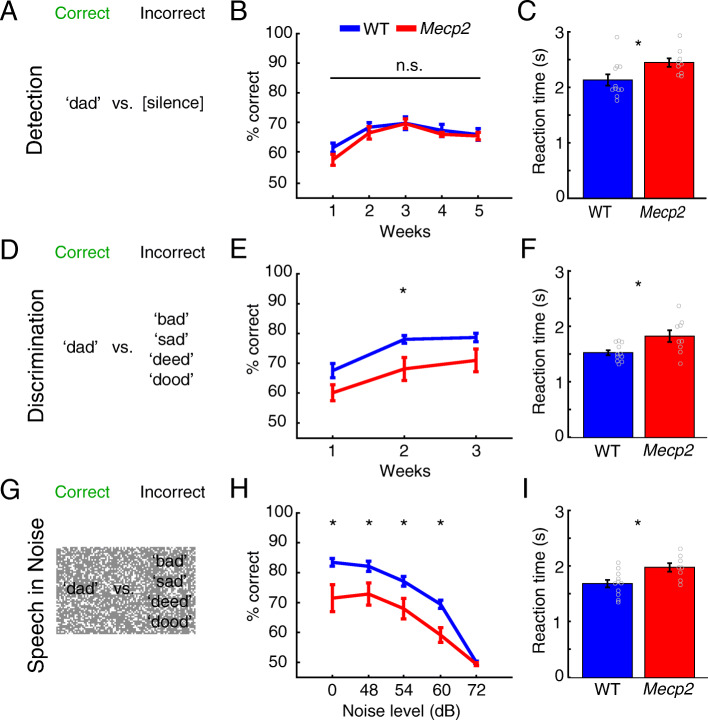
Speech discrimination performance is impaired in *Mecp2* rats. Target and non-target sounds for the **a** detection task, **d** speech discrimination task, and **g** speech in noise task. **b** Detection, a simple auditory behavior, was unimpaired in *Mecp2* rats compared to WT (*Mecp2 n* = 9, WT *n* = 11). However, *Mecp2* rats displayed significant impairments in the more challenging tasks: **e** speech discrimination in quiet (*Mecp2 n* = 9, WT *n* = 11) and **h** speech discrimination in varying levels of background noise (*Mecp2 n* = 8, WT *n* = 11). **c**, **f**, **i** *Mecp2* rats were significantly slower at responding to the target sound in all tasks. Circles depict individual subjects. Bars represent the mean and error bars indicate SEM across rats. The asterisk (*) denotes *p* < 0.05 across groups

Speech discrimination tasks and procedures were similar to previous studies [7–9]. Training sessions occurred twice a day for 1 h each, 5 days a week. Rats were first trained to press a lever or nose poke for a food reward until they reached a criteria of 100 independent responses for two sessions. Once this criteria was met, rats began training on a sound detection task. Rats were trained to respond to the target speech sound ‘dad’ within 3 s of sound presentation until they reached the criteria of ≥ 75% correct for 5 sessions, or until they reached 50 sessions of detection. Rats received a 45 mg food pellet reward (45 mg dustless precision pellet, BioServ Frenchtown, NJ) for a correct response to the target sound ‘dad’, and received a 6-s time out for an incorrect response to silence catch trials (Fig. 1a). Following this detection task, rats were trained on a speech discrimination task, in which they were required to respond to the target sound ‘dad’ and ignore the non-target sounds ‘bad’, ‘sad’, ‘deed’, and ‘dood’ (Fig. 1d). Each rat was trained on the discrimination task for 3 weeks. Rats then began training on a speech in noise task in which they were tasked to respond to the target sound ‘dad’, and ignore non-target sounds (‘bad’, ‘sad’, ‘deed’, and ‘dood’) during various levels of background noise (0, 48, 54, 60, and 72 dB) with the speech sounds presented at 60 dB (Fig. 1g). Each rat was trained on the speech in noise task for 2 weeks.

All speech sounds were spoken by a female native English speaker, as in previous studies [7–9]. Sounds were presented so that the loudest 100 ms of the vowel was 60 dB. The STRAIGHT vocoder was used to frequency shift all speech sounds up by one octave into the rat hearing range, while leaving all temporal information intact [19].

### Motor behavioral testing

**Fig. 2:**
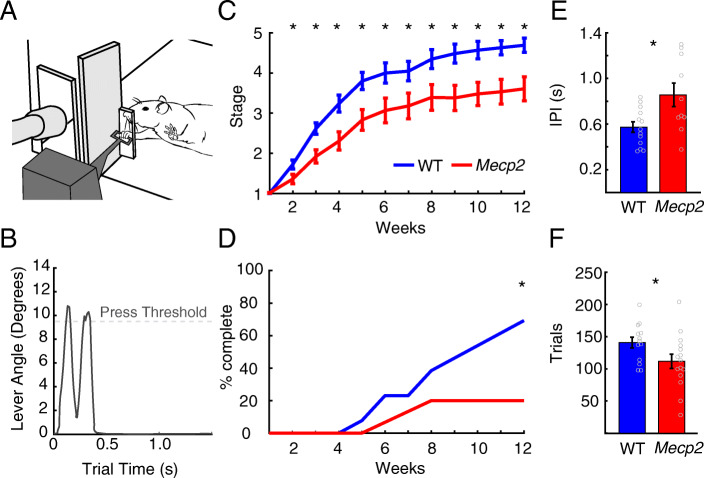
Skilled motor learning is impaired in *Mecp2* rats. **a** Illustration of a rat performing the lever pressing task. Rats were tasked with reaching out through a narrow slot in the cage and pressing a lever twice in rapid succession. **b** Representative data collected using the task, depicting a trial with a successful double press. **c** *Mecp2* rats were significantly slower to progress though training than WT (*Mecp2 n* = 15, WT *n* = 13). **d** A smaller portion of *Mecp2* rats completed the final stage of training within 12 weeks compared to WT rats (*Mecp2 n* = 15, WT *n* = 13). **e** *Mecp2* rats demonstrated significant reductions in lever pressing speed, as evidenced by an increase in the inter-press interval (IPI) (*Mecp2 n* = 10, WT *n* = 13). **f** Additionally, *Mecp2* rats completed significantly fewer trials than WT animals (*Mecp2 n* = 15, WT *n* = 13). Circles depict individual subjects. Bars represent the mean and error bars indicate SEM across rats. The asterisk (*) denotes *p* < 0.05 across groups

Rats were trained on a skilled motor task, as in previous studies [15–17, 23]. The task was an automated lever pressing task, in which the animal was required to learn to reach outside of a cage and press a lever twice in rapid succession. The behavior apparatus consisted of an acrylic cage, with a slot in the front right for access to a lever that was positioned 1 (inside) to 2 cm (outside) away from inside the edge of the chamber [15–17, 23] (Fig. 2a). Reward pellets were delivered to a receptacle in the front left side of the apparatus. A potentiometer was affixed to the lever to record the angle of the lever relative to the horizontal. The lever allows for up to a 13° depression. A lever press was defined as a deflection greater than 9.5°, followed by a release to at least 4.75°. A spring supported the lever, providing 28 g of resistance and allowing the lever to return to its horizontal resting position. An electronic controller board sampled the potentiometer position at 100 Hz and relayed the information to MotoTrak software the controlled the task criteria and collected data (MotoTrak, Vulintus, Louisville, CO).

Training sessions were 30 min in duration and occurred twice per day separated by at least 2 h, 5 days per week. Rats were first trained to press a lever that was positioned inside the apparatus. Throughout training, rats progressed through five stages, each requiring different criteria for trial success and consequent reward delivery, as detailed in Table 1. During the final stage, rats were rewarded for double pressing within a 500 ms window (Fig. 2b). Once the final stage was completed, rats underwent intracortical microstimulation (ICMS).

**Table 1:** Motor behavioral training stage parameters

Training stage	Hit time window (s)	Lever Location (cm)	Reward criteria	Criterion for advancement to next stage
Stage 1	2.0	− 1	Single lever press	30 pellets per session for 3 sessions
Stage 2	2.0	− 1	Release of first lever press	30 pellets per session for 3 sessions
Stage 3	2.0	− 1 ➔ 2	Release of first lever press	30 pellets per session for 3 sessions
Stage 4	0.5 - 2.0	2	Double lever press	30 pellets per session for 3 sessions
Stage 5	0.5	2	Double lever press	100 pellets and 65% hit rate per session for 3 sessions

### Intracortical microstimulation

The day after the final day of motor training, rats underwent ICMS to evaluate left motor cortex organization contralateral to the trained paw. Rats were anesthetized with ketamine hydrochloride (70 mg/kg, i.p.) and xylazine (5 mg/kg, i.p.), with supplementary doses given as needed to maintain anesthesia levels. Doxapram (20 mg/kg, i.p.) and glycopyrrolate (0.5 mg/kg, i.p.) were given to stabilize breathing and heart rate as needed. A small incision of the cistern magna was made to attenuate cortical swelling. A craniotomy and durotomy was performed to expose the left motor cortex. A tungsten electrode (0.1–1 MΩ) was inserted into the brain at a depth of 1.75 mm. Stimulation sites were then chosen at random on a grid with sites set 500 μm apart from each other. ICMS stimulation consisted of a 40 ms pulse train of 10 pulses.

ICMS procedures were conducted with two experimenters to ensure blinding to group and electrode location. The first experimenter placed the electrode and recorded data from each site. The second experimenter, blinded to the genotype of the rat and electrode position, delivered stimulations and classified movements. Stimulation was gradually increased from 20 to 250 μA, or until a movement was observed. The stimulation amplitude at which a movement was first seen was documented as the threshold. If no movement was seen at 250 μA, then that site was recorded as no response. Movements were classified as proximal forelimb, distal forelimb, head, or hindlimb. Cortical area was calculated by multiplying the number of sites eliciting a response by the area surrounding a site (0.25 mm^2^).

### Social behavior tests

To assess social behavior, 9 *Mecp2* rats and 9 WT littermates underwent a sociability and social novelty preference task. The behavioral arena was a rectangular box 70 cm in length × 30 cm wide × 50 cm depth. The apparatus was divided into three chambers. The two outside chambers each contained a wire cage. The experimental animal was placed inside the middle chamber and allowed to freely explore for 5 min to habituate to the apparatus. To test sociability, an unfamiliar rat that had no prior contact was then placed into one of the wired cages, and the experimental rat freely explored for 10 min. Sociability was defined as the amount of time the experimental rat spent in the chamber with the unfamiliar rat. The sociability index (time spent with the unfamiliar rat − time spent in the empty chamber) ÷ (time spent with the unfamiliar rat + time spent in the empty chamber) was used to indicate a preference to interact with or avoid the rat. To test social novelty, a second unfamiliar rat was placed in the wired cage in the chamber that had previously been empty. The experimental rat was allowed to freely roam for another 10 min. Social novelty was defined as how much time was spent with the novel rat compared to the now familiar rat. The social novelty index (time spent with the novel rat − time spent with the familiar rat) ÷ (time spent with the novel rat + time spent with the familiar rat) was used to indicate a preference to interact with the novel rat over the familiar rat. The rats used during the sociability and social novelty tests were of the same age and sex as the experimental rats.

### Standard behavioral assessments

To assess repetitive behaviors, 9 *Mecp2* rats and 9 WT littermates underwent a marble burying test. Rats were habituated to the novel bedding (BioFresh nitrocellulose comfort bedding). Rats were then placed into a new cage with 15 marbles for 10 min. The number of marbles buried was recorded.

Nine *Mecp2* rats and 9 WT littermates also underwent a spontaneous alternation task. Rats were placed in a plus maze and were allowed to freely explore for 10 min. A spontaneous alternation occurred when a rat entered all arms within five consecutive entries. The percentage of spontaneous alternation was calculated by dividing the number of alternations by the number of possible alternations (number of alternations) ÷ (number of total arm entries − 4) × 100.

### Statistical analysis

Statistical analysis was performed with MATLAB software. Speech discrimination performance was measured as percent correct, determined by the average of correct responses to the target speech sound and correct rejections to the non-target speech sounds. Performance on the speech in noise task was taken from the first week of training. The average reaction time to respond to the target sound was also assessed. Two-way repeated measure ANOVAs were used to analyze discrimination performance between experimental groups as well as discrimination performance over time. Post hoc unpaired *t* tests were used to determine statistical significance. A repeated measure general linear model was used to analyze speech in noise performance between groups and noise levels. Post hoc pairwise comparisons were Bonferroni-corrected for each noise level. Behavioral analysis of motor function included average training stage progression, number of trials per session, and lever inter-press interval (IPI). Significant differences for average stage progression were determined using the Wilcoxon rank-sum test. Mean IPI was calculated from the fourth and fifth stages. Significant differences in behavior measures were determined using unpaired *t* tests. The chi-square test was used to determine significance in the portion of rats that completed the study. To assess ICMS data, an unpaired *t* test was used to determine significance in movement representation size, total map size, and average thresholds. Measures for the behavioral assays include the number of marbles buried, sociability, social novelty, and percent alternations. Unpaired *t* tests were used to determine group differences in behavioral assays. All data is reported as mean +/− standard error of the mean (SEM).

## Results

A number of studies document alterations in auditory processing in individuals with Rett syndrome [1, 10, 18, 27, 28] and animal models [9, 12, 20]. We sought to evaluate the acquisition and performance on a speech discrimination task in *Mecp2* rats to confirm previous findings [9]. *Mecp2* and WT rats were initially trained to respond to presentation of the speech sound ‘dad’. Detection of the speech sound, calculated as the percentage of trials in which rats correctly responded to the target sound, did not significantly improve in either groups over the course of 5 weeks of training (Fig. 1b; two-way repeated measures ANOVA, *F*(4, 28) = 1.3288, *p* = 0.238). No differences in performance were observed in between groups on this simple task (Fig. 1b; two-way repeated measures ANOVA, effect of group; *F*(1, 7) = 0.025, *p* = 0.878). A significantly slower reaction time was observed in the *Mecp2* group compared to the WT control group (Fig. 1c; *Mecp2* 2.45 ± 0.07 s, WT 2.13 ± 0.1 s, Unpaired *t* test, *p* = 0.029). These findings are consistent with previous studies and suggest that gross auditory processing is generally intact in *Mecp2* rodents [9, 12, 20].

We next assessed performance in these rats on a more challenging auditory task that requires discrimination of the target sound from a number of similar distractor sounds, either in a quiet environment or in the presence of background noise. *Mecp2* rats demonstrated significantly impaired performance compared to WT in the absence of background noise over the course of 3 weeks of testing (Fig. 1e; two-way repeated measures ANOVA, effect of group; *F*(1, 18) = 5.99, *p* = 0.025). Post hoc analysis revealed that *Mecp2* rats performed significantly worse in week 2 of discrimination training and a trend towards worse performance on weeks 1 and 3 (unpaired *t* test, week 1: *p* = 0.054, *t* = 2.05; week 2: *p* = 0.0172, *t* = 2.62; week 3 *p* = 0.055, *t* = 2.04). The presence of background noise significantly degraded discrimination performance in both groups (Fig. 1h; repeated measures general linear model, effect of noise level; *F*(4, 14) = 50.66, *p* = 3.55 × 10^ − 8). However, *Mecp2* rats performed significantly worse than WT in multiple levels of background noise (Fig. 1h; repeated measures general linear model, effect of noise level*group; *F*(4, 14) = 3.392, *p* = 0.039). Post hoc analysis revealed that *Mecp2* rats performed significantly worse when 0–60 dB background noise was present (pairwise comparisons, 0 dB: *F*(1, 17) = 9.57, *p* = 0.007; 48 dB: *F*(1, 17) = 6.97, *p* = 0.017; 54 dB: *F*(1, 17) = 7.82, *p* = 0.012; 60 dB: *F*(1, 17) = 16.68, *p* = 0.001; 72 dB: *F*(1, 17) = 1.30, *p* = 0.268). Consistent with reduced performance, *Mecp2* rats exhibited significantly slower reaction times (Fig. 1f, i; unpaired *t* test, discrimination: *Mecp2*: 1.82 ± 0.11 s, WT: 1.52 ± 0.04 s, *p* = 0.013; speech in noise: *Mecp2*: 1.99 ± 0.08 s, WT: 1.69 ± 0.07 s, *p* = 0.012). To confirm that impaired performance is not due to motor deficits that slow reaction time, we extended the hit window to 6 s for *Mecp2* rats and reanalyzed discrimination performance. Even with this prolonged hit window, *Mecp2* rats demonstrated significantly impaired discrimination compared to WT over the course of 3 weeks (6 s hit window: two-way repeated measures ANOVA, effect of group; *F*(1, 18) = 5.96, *p* = 0.025). These findings indicate that impaired performance is not likely due to motor deficits that may slow response rate, but rather can be ascribed to incorrect responses. These findings indicate that *Mecp2* rats exhibit deficits on a complex auditory task that are not apparent in a simple task.

Previous studies report modest deficits in motor function in *Mecp2* heterozygous or KO animal models [3, 13, 24, 26, 29, 31, 32], but these deficits largely fail to replicate the substantial motor dysfunction observed in Rett patients. Given the appearance of deficits on a challenging auditory task, we next sought to characterize acquisition and performance of *Mecp2* rats on a skilled forelimb motor task. *Mecp2* and WT rats underwent training on a task that requires the rats to reach their forelimb through a narrow slot in the cage and press a lever twice in rapid succession to receive a food reward. The training was staged to become progressively more challenging as performance improved, such that longer reaches and faster presses were required (Table 1). *Mecp2* rats demonstrated significantly slower progression through training stages than WT animals over training, indicative of an impairment in skilled motor learning (Fig. 2c; Wilcoxon rank-sum test, weeks 2–12, *p* < 0.05). Consequently, a smaller proportion of *Mecp2* rats completed the final stage of training within 12 weeks compared to WT (Fig. 2d; *Mecp2*: 3 of 15 rats, WT: 9 of 13 rats, *χ*^2^ test, *χ*^2^ = 6.89, *p* = 0.009). *Mecp2* rats displayed a significantly slower interval between successive lever presses than WT (Fig. 2e; *Mecp2*: 0.86 ± 0.1 s, WT: 0.57 ± 0.04 s, unpaired *t* test, *p* = 0.013). Additionally, *Mecp2* rats performed fewer trials during behavioral training sessions, consistent with previous reports of motor hypoactivity [3, 13, 24, 26, 29, 31, 32] (Fig. 2f; *Mecp2*: 114.29 ± 11.27, WT: 144.19 ± 8.61, unpaired *t* test, *p* = 0.049). Together, these results indicate an impairment in skilled motor function in *Mecp2* rats.

**Fig. 3:**
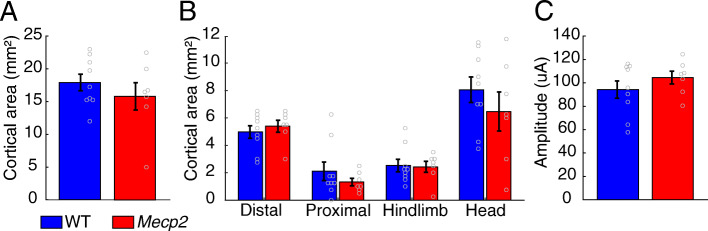
Gross organization of cortical motor circuits is preserved in *Mecp2* rats. **a** Total cortical movement area and **b** cortical area of individual movement representations were unaltered in *Mecp2* rats. **c** Stimulation threshold to evoke movements was also not altered in *Mecp2* rats. Circles depict individual subjects (WT *n* = 9, *Mecp2 n* = 7). Bars represent the mean and error bars indicate SEM across rats

We next sought to explore whether large scale changes in cortical circuits could explain motor performance deficits in *Mecp2* rats. To do so, we conducted ICMS mapping to document movement representations in the motor cortex after the conclusion of motor training. No differences in overall area of the motor cortex or the size of individual movement representations were observed between *Mecp2* and WT rats (Fig. 3a, unpaired *t* test, overall area: *Mecp2*: 15.82 ± 2.09 mm^2^, WT: 17.92 ± 1.25 mm^2^, *p* = 0.38; Fig. 3b, distal forelimb area: *Mecp2*: 5.39 ± 0.44 mm^2^, WT: 4.97 ± 0.45 mm^2^, *p* = 0.53; proximal forelimb area: *Mecp2*: 1.32 ± 0.27 mm^2^, WT: 2.11 ± 0.68 mm^2^, *p* = 0.34; hindlimb area: *Mecp2*: 2.43 ± 0.4 mm^2^, WT: 2.53 ± 0.45 mm^2^, *p* = 0.88; head area: *Mecp2*: 6.46 ± 1.43 mm^2^, WT: 8.06 ± 0.94 mm^2^, *p* = 0.35). Additionally, the stimulation threshold to evoke a movement was similar between groups (Fig. 3c; *Mecp2*: 104.52 ± 5.47 μA, WT: 94.19 ± 7.38 μA, unpaired *t* test, *p* = 0.3). The absence of gross changes in cortical movement representations suggests that motor dysfunction may arise from changes in synaptic function in motor networks, rather than large-scale changes in corticospinal connectivity.

**Fig. 4:**
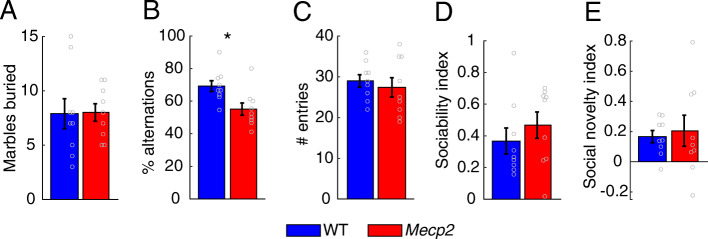
*Mecp2* rats display some cognitive impairments. **a** The number of marbles buried was not significantly different between *Mecp2* rats and WT. **b** *Mecp2* rats exhibit significantly fewer spontaneous alternations compared to WT. **c** No difference in the number of entries was observed. **d**, **e** Additionally, no difference in sociability or social novelty was observed between groups. Circles depict individual subjects (WT *n* = 9, *Mecp2 n* = 9). Bars represent the mean and error bars indicate SEM across rats. The asterisk (*) denotes *p* < 0.05 across groups

Previous studies have documented social and cognitive deficits in *Mecp2* rodents, so we sought to corroborate those findings [6, 11, 26, 30–32]. *Mecp2* and WT rats underwent a social interaction task in which we measured their sociability and social novelty. No difference in sociability or social novelty was observed between groups (Fig. 4d, e; unpaired *t* test, sociability index: *Mecp2*: 0.47 ± 0.08, WT: 0.36 ± 0.08, *p* = 0.4; social novelty index: *Mecp2*: 0.2 ± 0.1, WT: 0.17 ± 0.04, *p* = 0.72). *Mecp2* and WT rats also exhibited comparable behavior on a marble burying task (Fig. 4a; *Mecp2*: 8 ± 0.8 marbles, WT: 7.89 ± 1.37 marbles, unpaired *t* test, *p* = 0.94). In a spontaneous alternation task, *Mecp2* rats have significantly less spontaneous alternations compared to WT controls (Fig. 4b; *Mecp2*: 55% ± 3.77, WT: 69% ± 3.31, unpaired *t* test, *p* = 0.012). Spontaneous alternation is also used to measure anxiety or locomotor activity; however, the number of entries to each arm was not different (Fig. 4c; *Mecp2*: 27.44 ± 2.36 entries, WT: 29 ± 1.52 entries, unpaired t test, *p* = 0.59), suggesting that locomotor activity or anxiety did not drive any spontaneous alternation behavior.

## Discussion

The present study provides a novel characterization of complex skill learning in *Mecp2* rats. We report that heterozygous *Mecp2* rats display slower acquisition and reduced performance on an auditory discrimination task than WT littermates. Similarly, *Mecp2* rats exhibit impaired learning rates and worse performance on a skilled forelimb motor task compared to WT. Together, these findings illustrate skilled performance deficits that are novel and consistent with clinical manifestation.

We find that *Mecp2* rats were impaired during a more challenging auditory task, corroborating a previous report [9]. These findings are consistent with clinical evidence that while individuals with Rett syndrome generally have normal gross auditory processing, higher levels of auditory processing are often degraded [1, 9, 12, 18, 20, 27, 28]. This is also supported by evidence of higher hearing thresholds and decreased response strength to low frequency sounds in the primary auditory cortex of *Mecp2* rats [9]. Additionally, we found that *Mecp2* rats were slower at responding to the target sound on all speech discrimination tasks. Both individuals with Rett syndrome and *Mecp2* animal models exhibit delayed cortical responses to auditory stimuli [1, 9, 10, 12, 20]. This degraded auditory processing likely contributes to the slower reaction times to auditory stimuli observed in the current study.

Given that *Mecp2* rats were impaired during a more challenging auditory task, we sought to characterize performance on a skilled forelimb motor task. Overall, *Mecp2* rats had a significant impairment in skilled motor function compared to WT controls. In the current study, the reduced forelimb movement speed, as assessed by longer latencies between presses on the task, are consistent with deficits in motor performance in *Mecp2* rats [5, 31]. Additionally, *Mecp2* rats initiated fewer trials during behavioral testing sessions, consistent with motor hypoactivity previously documented with the open field and rotarod tasks [3, 13, 24, 26, 29, 31, 32]. The impairments in motor learning and skilled motor function reported here are consistent with clinical observations. Moreover, these findings expand the utility of the transgenic rat model and suggest that the model may be useful as a framework to evaluate interventions aimed at improving motor function.

Studies have documented impaired motor cortex plasticity in both *Mecp2* rodent models [21, 22] and Rett syndrome patients [2], which may contribute to motor dysfunction. Based on this data and our observation of deficits in skilled motor function, we sought to explore whether large-scale changes in cortical circuits could explain poor motor performance in *Mecp2* rats. However, no differences in the cortical movement representation area or stimulation threshold to evoke movements were observed. The technique used to evaluate motor function in this study, intracortical microstimulation, largely provides a coarse evaluation of direct corticospinally evoked movements. Similar to the auditory system, our findings indicate that motor circuits are largely preserved in *Mecp2* heterozygous rats, and the motor dysfunction likely reflects changes in synaptic plasticity or dysfunction in higher processing areas. Future studies directed at examining finer-scale synaptic changes in motor networks may provide more insight into skilled motor dysfunction.

Along with severe auditory and motor dysfunction, Rett syndrome patients exhibit cognitive and social deficits. This has been modeled in various rodent models of Rett syndrome [6, 11, 26, 30–32]. We sought to corroborate some of these findings in our *Mecp2* female rat model. To do so, *Mecp2* and WT rats underwent a social interaction task, a marble burying task, and a spontaneous alternation task to assess sociability and social preference, repetitive behaviors, and working memory, respectively. While we did not observe differences in social or repetitive behaviors, we did observe a working memory impairment, indicated by a reduction in percent alternation in the spontaneous alternation task. This finding is consistent with a previous study that observed working memory impairments in a novel *Mecp2* male mouse model [11]. Conversely, we did not observe abnormal social behavior or repetitive movements. This difference may arise from the fact that female *Mecp2* rodents used in the present study exhibit a less severe phenotype compared to male rodents [24, 26]. Additionally, some studies have suggested that social behavior may improve over time in Rett syndrome patients [33], which may explain the lack of abnormal social behavior in the current study. A previous study documented abnormal social behavior in juvenile female *Mecp2* rats [31], while the current study used adult *Mecp2* rats. Future studies evaluating social behavior at several timepoints may provide more insight to the manifestation and time course of abnormal social behavior in *Mecp2* rats.

Several studies have successfully modeled *MECP2* dysfunction and Rett syndrome-like phenotypes in *Mecp2* mice [12, 13, 20–22, 26, 29]. The *Mecp2* rat model used in the present study provides the opportunity for more complex behavioral analysis that may offer more insight into the pathology of Rett syndrome. Additionally, our experiments were conducted in adult *Mecp2* heterozygous females, which is more analogous to heterozygous mutations found in girls with Rett syndrome. A recent preceding study highlights the utility of the female *Mecp2* rat model, using the model to identify psychomotor regression similarly seen in Rett syndrome girls [31].

A number of limitations of this study merit consideration. While the presented data provides initial evidence of skilled learning deficits in auditory and motor tasks in *Mecp2* rats, rodent models fail to capture the complexity of skilled learning in humans. Future studies that expand evaluation to a broader range of tasks will provide a more comprehensive assessment of skilled learning in this model. In the current study, we observed significantly slower reaction times in *Mecp2* rats, consistent with reduced performance on the speech discrimination task. Based on the present data, we cannot exclude the possibility that slower reaction time arise from abnormal motor function. However, given that *Mecp2* rodents have generally intact motor function but abnormal auditory cortical responses [9, 12, 20], we believe that the speech discrimination impairment is due to atypical cortical auditory processing. Future studies should incorporate recording of neural responses in the brainstem and the auditory cortex in conjunction with behavioral discrimination to examine the contribution of auditory processing. One of the central findings of the present study is the observation of impaired motor learning and performance on a skilled task. The current study also found that *Mecp2* rats performed fewer trials during motor behavioral training sessions, which is consistent with previous reports of motor hypoactivity with the open field and rotarod tasks [3, 13, 24, 26, 29, 31, 32]. It is possible that impaired motor skills could arise from lack of motivation or general muscle weakness. Preliminary results from a previous study indicate potential muscle weakness in *Mecp2* females, but the magnitude of this effect is unclear [24]. Moreover, recent evidence found that impairments in motor cortex plasticity correlates with motor deficit in individuals with Rett syndrome, suggesting that central changes contribute to motor dysfunction [2]. Future studies are needed to delineate the contributions of central and peripheral mechanisms on motor dysfunction.

## Conclusions

The development of interventional strategies to improve the quality of life of individuals with Rett syndrome is of clinical importance. Characterization of more complex behaviors known to be altered in Rett syndrome could expand the utility of animal models. The current study characterizes skilled motor learning, replicates previous work evaluating auditory discrimination performance in female *Mecp2* rats, and establishes a framework for future studies to evaluate the effect of potential interventions on complex behavioral tasks.

## Supplementary information

**Additional file 1.** Task assignment for each animal.

## Data Availability

The datasets used during the current study are available from the corresponding author on reasonable request.

## Abbreviations

*Mecp2*: Methyl CpG binding protein 2
WT: Wild-type
ICMS: Intracortical microstimulation

## References

[1] Bader, G. G., Witt-Engerström, I., & Hagberg, B. (1989). Neurophysiological findings in the Rett syndrome, II: visual and auditory brainstem, middle and late evoked responses. Brain Dev, 11(2), 110–114. 10.1016/S0387-7604(89)80078-6. 10.1016/s0387-7604(89)80078-6 2712233

[2] Bernardo, P., Cobb, S., Coppola, A., Tomasevic, L., Di Lazzaro, V., Bravaccio, C., Manganelli, F., & Dubbioso, R. (2020). Neurophysiological signatures of motor impairment in patients with Rett syndrome. Ann Neurol, 763–773. 10.1002/ana.25712. 10.1002/ana.25712 32129908

[3] Bhattacherjee, A., Winter, M., Eggimann, L., Mu, Y., Gunewardena, S., Liao, Z., Christianson, J., & Smith, P. (2017). Motor, somatosensory, viscerosensory and metabolic impairments in a heterozygous female rat model of Rett syndrome. Int J Mol Sci, 19(1), 97. 10.3390/ijms19010097. 10.3390/ijms19010097 PMC5796047 29286317

[4] Chahrour, M., & Zoghbi, H. Y. (2007). The story of Rett syndrome: from clinic to neurobiology. In Neuron (Vol. 56, Issue 3, pp. 422–437). 10.1016/j.neuron.2007.10.001 17988628

[5] De Filippis, B., Musto, M., Altabella, L., Romano, E., Canese, R., & Laviola, G. (2015). Deficient purposeful use of forepaws in female mice modelling Rett syndrome. Neural Plasticity, 2015. 10.1155/2015/326184. 10.1155/2015/326184 PMC4491574 26185689

[6] De Filippis, B., Nativio, P., Fabbri, A., Ricceri, L., Adriani, W., Lacivita, E., Leopoldo, M., Passarelli, F., Fuso, A., & Laviola, G. (2014). Pharmacological stimulation of the brain serotonin receptor 7 as a novel therapeutic approach for rett syndrome. Neuropsychopharmacology, 39(11), 2506–2518. 10.1038/npp.2014.105. 10.1038/npp.2014.105 PMC4207333 24809912

[7] Engineer, C. T., Perez, C. A., Carraway, R. S., Chang, K. Q., Roland, J. L., & Kilgard, M. P. (2014). Speech training alters tone frequency tuning in rat primary auditory cortex. Behav Brain Res, 258, 166–178. 10.1016/j.bbr.2013.10.021. 10.1016/j.bbr.2013.10.021 PMC3886187 24344364

[8] Engineer, C. T., Perez, C. A., Chen, Y. H., Carraway, R. S., Reed, A. C., Shetake, J. A., Jakkamsetti, V., Chang, K. Q., & Kilgard, M. P. (2008). Cortical activity patterns predict speech discrimination ability. Nat Neurosci, 11(5), 603–608. 10.1038/nn.2109. 10.1038/nn.2109 PMC2951886 18425123

[9] Engineer, C. T., Rahebi, K. C., Borland, M. S., Buell, E. P., Centanni, T. M., Fink, M. K., Im, K. W., Wilson, L. G., & Kilgard, M. P. (2015). Degraded neural and behavioral processing of speech sounds in a rat model of Rett syndrome. Neurobiol Dis, 83(6), 26–34. 10.1016/j.nbd.2015.08.019. 10.1016/j.nbd.2015.08.019 PMC4674323 26321676

[10] Foxe, J. J., Burke, K. M., Andrade, G. N., Djukic, A., Frey, H.-P., & Molholm, S. (2016). Automatic cortical representation of auditory pitch changes in Rett syndrome. J Neurodev Disord, 8(1), 34. 10.1186/s11689-016-9166-5. 10.1186/s11689-016-9166-5 PMC5009506 27594924

[11] Gandaglia, A., Brivio, E., Carli, S., Palmieri, M., Bedogni, F., Stefanelli, G., Bergo, A., Leva, B., Cattaneo, C., Pizzamiglio, L., Cicerone, M., Bianchi, V., Kilstrup-Nielsen, C., D’Annessa, I., Di Marino, D., D’Adamo, P., Antonucci, F., Frasca, A., & Landsberger, N. (2019). A novel Mecp2 Y120D knock-in model displays similar behavioral traits but distinct molecular features compared to the Mecp2-null mouse implying precision medicine for the treatment of Rett syndrome. Mol Neurobiol, 56(7), 4838–4854. 10.1007/s12035-018-1412-2. 10.1007/s12035-018-1412-2 30402709

[12] Goffin, D., Allen, M., Zhang, L., Amorim, M., Wang, I.-T. J., Reyes, A.-R. S., Mercado-Berton, A., Ong, C., Cohen, S., Hu, L., Blendy, J. a, Carlson, G. C., Siegel, S. J., Greenberg, M. E., & Zhou, Z. (2011). Rett syndrome mutation MeCP2 T158A disrupts DNA binding, protein stability and ERP responses. Nat Neurosci, 15(2), 274–283. 10.1038/nn.2997. 10.1038/nn.2997 PMC3267879 22119903

[13] Guy, J., Hendrich, B., Holmes, M., Martin, J. E., & Bird, a. (2001). A mouse Mecp2-null mutation causes neurological symptoms that mimic Rett syndrome. Nat Genet, 27(3), 322–326. 10.1038/85899. 10.1038/85899 11242117

[14] Hagberg, B., Witt-Engerström, I., Opitz, J. M., & Reynolds, J. F. (1986). Rett syndrome: a suggested staging system for describing impairment profile with increasing age towards adolescence. Am J Med Genet, 25(S1), 47–59. 10.1002/ajmg.1320250506. 10.1002/ajmg.1320250506 3087203

[15] Hays, S. A., Khodaparast, N., Sloan, A. M., Fayyaz, T., Hulsey, D. R., Ruiz, A. D., Pantoja, M., Kilgard, M. P., & Rennaker, R. L. (2013). The bradykinesia assessment task: an automated method to measure forelimb speed in rodents. J Neurosci Methods, 214(1), 52–61. 10.1016/j.jneumeth.2012.12.022. 10.1016/j.jneumeth.2012.12.022 23353133

[16] Hulsey, D. R., Hays, S. A., Khodaparast, N., Ruiz, A., Das, P., Rennaker, R. L., & Kilgard, M. P. (2016). Reorganization of motor cortex by vagus nerve stimulation requires cholinergic innervation. Brain Stimulation, 9(2), 174–181. 10.1016/j.brs.2015.12.007. 10.1016/j.brs.2015.12.007 PMC4789078 26822960

[17] Hulsey, D. R., Shedd, C. M., Sarker, S. F., Kilgard, M. P., & Hays, S. A. (2019). Norepinephrine and serotonin are required for vagus nerve stimulation directed cortical plasticity. Exp Neurol, 320(February), 112975. 10.1016/j.expneurol.2019.112975. 10.1016/j.expneurol.2019.112975 PMC6708444 31181199

[18] Kálmánchey, R. (1990). Evoked potentials in the rett syndrome. Brain Dev, 12(1), 73–76. 10.1016/S0387-7604(12)80181-1. 10.1016/s0387-7604(12)80181-1 2344031

[19] Kawahara, H. (1997). Speech representation and transformation using adaptive interpolation of weighted spectrum: vocoder revisited. 1997 IEEE International Conference on Acoustics, Speech, and Signal Processing, 2, 1303–1306. 10.1109/ICASSP.1997.596185.

[20] Liao, W., Gandal, M. J., Ehrlichman, R. S., Siegel, S. J., & Carlson, G. C. (2012). MeCP2+/− mouse model of RTT reproduces auditory phenotypes associated with Rett syndrome and replicate select EEG endophenotypes of autism spectrum disorder. Neurobiol Dis, 46(1), 88–92. 10.1016/j.nbd.2011.12.048. 10.1016/j.nbd.2011.12.048 PMC3299908 22249109

[21] Morello, N., Schina, R., Pilotto, F., Phillips, M., Melani, R., Plicato, O., Pizzorusso, T., Pozzo-Miller, L., & Giustetto, M. (2018). Loss of Mecp2 causes atypical synaptic and molecular plasticity of parvalbumin-expressing interneurons reflecting rett syndrome-like sensorimotor defects. Eneuro, 5(October), ENEURO.0086-18.2018. 10.1523/ENEURO.0086-18.2018. 10.1523/ENEURO.0086-18.2018 PMC6153339 30255129

[22] Moretti P, Levenson JM, Battaglia F, Atkinson R, Teague R, Antalffy B (2006). Learning and memory and synaptic plasticity are impaired in a mouse model of Rett syndrome. J Neurosci.

[23] Morrison, R. A., Hulsey, D. R., Adcock, K. S., Rennaker, R. L., Kilgard, M. P., & Hays, S. A. (2019). Vagus nerve stimulation intensity influences motor cortex plasticity. Brain Stimulation, 12(2), 256–262. 10.1016/j.brs.2018.10.017. 10.1016/j.brs.2018.10.017 PMC6347516 30409712

[24] Patterson, K. C., Hawkins, V. E., Arps, K. M., Mulkey, D. K., & Olsen, M. L. (2016). MeCP2 deficiency results in robust Rett-like behavioural and motor deficits in male and female rats. Hum Mol Genet, 25(15), 3303–3320. 10.1093/hmg/ddw179. 10.1093/hmg/ddw179 PMC5179928 27329765

[25] Samaco, R. C., Fryer, J. D., Ren, J., Fyffe, S., Chao, H. T., Sun, Y., Greer, J. J., Zoghbi, H. Y., & Neul, J. L. (2008). A partial loss of function allele of methyl-CpG-binding protein 2 predicts a human neurodevelopmental syndrome. Hum Mol Genet, 17(12), 1718–1727. 10.1093/hmg/ddn062. 10.1093/hmg/ddn062 PMC2666042 18321864

[26] Samaco, R. C., Mcgraw, C. M., Ward, C. S., Sun, Y., Neul, J. L., & Zoghbi, H. Y. (2013). Female Mecp2+/− mice display robust behavioral deficits on two different genetic backgrounds providing a framework for pre-clinical studies. Hum Mol Genet, 22(1), 96–109. 10.1093/hmg/dds406. 10.1093/hmg/dds406 PMC3522402 23026749

[27] Stach, B. A., Stoner, W. R., Smith, S. L., & Jerger, J. F. (1994). Auditory evoked potentials in Rett syndrome. J Am Acad Audiol, 5(3), 226–230. 10.3171/JNS/2008/108/5/0950. 8075419

[28] Stauder, J. E. A., Smeets, E. E. J., van Mil, S. G. M., & Curfs, L. G. M. (2006). The development of visual and auditory processing in Rett syndrome: an ERP study. Brain Dev, 28(8), 487–494. 10.1016/j.braindev.2006.02.011. 10.1016/j.braindev.2006.02.011 16647236

[29] Stearns, N. A., Schaevitz, L. R., Bowling, H., Nag, N., Berger, U. V., & Berger-Sweeney, J. (2007). Behavioral and anatomical abnormalities in Mecp2 mutant mice: a model for Rett syndrome. Neuroscience, 146(3), 907–921. 10.1016/j.neuroscience.2007.02.009. 10.1016/j.neuroscience.2007.02.009 17383101

[30] Ure, K., Lu, H., Wang, W., Ito-Ishida, A., Wu, Z., He, L. J., Sztainberg, Y., Chen, W., Tang, J., & Zoghbi, H. Y. (2016). Restoration of Mecp2 expression in GABAergic neurons is sufficient to rescue multiple disease features in a mouse model of Rett syndrome. ELife, 5(JUN2016), 1–21. 10.7554/eLife.14198. 10.7554/eLife.14198 PMC4946897 27328321

[31] Veeraragavan, S., Wan, Y. W., Connolly, D. R., Hamilton, S. M., Ward, C. S., Soriano, S., Pitcher, M. R., McGraw, C. M., Huang, S. G., Green, J. R., Yuva, L. A., Liang, A. J., Neul, J. L., Yasui, D. H., LaSalle, J. M., Liu, Z., Paylor, R., & Samaco, R. C. (2015). Loss of MeCP2 in the rat models regression, impaired sociability and transcriptional deficits of Rett syndrome. Hum Mol Genet, 25(15), 3284–3302. 10.1093/hmg/ddw178. 10.1093/hmg/ddw178 PMC5179927 27365498

[32] Wu, Y., Zhong, W., Cui, N., Johnson, C. M., Xing, H., Zhang, S., & Jiang, C. (2016). Characterization of Rett syndrome-like phenotypes in Mecp2-knockout rats. J Neurodev Disord, 8(1), 23. 10.1186/s11689-016-9156-7. 10.1186/s11689-016-9156-7 PMC4910223 27313794

[33] Zappella, M., Meloni, I., Longo, I., Canitano, R., Hayek, G., Rosaia, L., Mari, F., & Renieri, A. (2003). Study ofMECP2 gene in Rett syndrome variants and autistic girls. Am J Med Genet, 119B(1), 102–107. 10.1002/ajmg.b.10070. 10.1002/ajmg.b.10070 12707946

